# Host Factor SAMHD1 Restricts DNA Viruses in Non-Dividing Myeloid Cells

**DOI:** 10.1371/journal.ppat.1003481

**Published:** 2013-06-27

**Authors:** Joseph A. Hollenbaugh, Peter Gee, Jonathon Baker, Michele B. Daly, Sarah M. Amie, Jessica Tate, Natsumi Kasai, Yuka Kanemura, Dong-Hyun Kim, Brian M. Ward, Yoshio Koyanagi, Baek Kim

**Affiliations:** 1 Department of Microbiology and Immunology, University of Rochester, Rochester, New York, United States of America; 2 Center for Drug Discovery, The Department of Pediatrics, Emory University, Atlanta, Georgia, United States of America; 3 Laboratory of Viral Pathogenesis, Institute for Virus Research, Kyoto University, Kyoto, Japan; 4 Department of Pharmacy, Kyung-Hee University, Seoul, South Korea; University of Pennsylvania School of Medicine, United States of America

## Abstract

SAMHD1 is a newly identified anti-HIV host factor that has a dNTP triphosphohydrolase activity and depletes intracellular dNTP pools in non-dividing myeloid cells. Since DNA viruses utilize cellular dNTPs, we investigated whether SAMHD1 limits the replication of DNA viruses in non-dividing myeloid target cells. Indeed, two double stranded DNA viruses, vaccinia and herpes simplex virus type 1, are subject to SAMHD1 restriction in non-dividing target cells in a dNTP dependent manner. Using a thymidine kinase deficient strain of vaccinia virus, we demonstrate a greater restriction of viral replication in non-dividing cells expressing SAMHD1. Therefore, this study suggests that SAMHD1 is a potential innate anti-viral player that suppresses the replication of a wide range of DNA viruses, as well as retroviruses, which infect non-dividing myeloid cells.

## Author Summary

Various viral pathogens such as HIV-1, herpes simplex virus (HSV) and vaccinia virus infect terminally-differentiated/non-dividing macrophages during the course of viral pathogenesis. Unlike dividing cells, non-dividing cells lack chromosomal DNA replication, do not enter the cell cycle, and harbor very low levels of cellular dNTPs, which are substrates of viral DNA polymerases. A series of recent studies revealed that the host protein SAMHD1 is dNTP triphosphohydrolase, which contributes to the poor dNTP abundance in non-dividing myeloid cells, and restricts proviral DNA synthesis of HIV-1 and other lentiviruses in macrophages, dendritic cells, and resting T cells. In this report, we demonstrate that SAMHD1 also controls the replication of large dsDNA viruses: vaccinia virus and HSV-1, in primary human monocyte-derived macrophages. SAMHD1 suppresses the replication of these DNA viruses to an even greater extent in the absence of viral genes that are involved in dNTP metabolism such as thymidine kinase. Therefore, this study supports that dsDNA viruses evolved to express enzymes necessary to increase the levels of dNTPs as a mechanism to overcome the restriction induced by SAMHD1 in myeloid cells.

## Introduction

It is becoming increasingly evident that host cells employ metabolic regulatory mechanisms in order to restrict the life cycle of pathogens [1], [2], [3], [4]. The recent discovery of sterile alpha motif (SAM) domain and histidine-aspartic (HD) domain-containing protein 1 (SAMHD1) has contributed to our understanding of the metabolic regulation of deoxynucleoside triphosphates (dNTPs), the substrate for cellular DNA polymerases to synthesize and repair host DNA. SAMHD1 expression limits proviral DNA synthesis in lentiviruses particularly in non-dividing myeloid cells such as macrophages and dendritic cells (DCs) [5], [6], [7], [8]. SAMHD1 is a dNTPs triphosphohydrolase, and functions by hydrolyzing dNTPs into dNs and triphosphates [9], [10], thus leading to the reduction of cellular dNTP concentrations [5], [6]. This in turn can impact the kinetics of cellular, viral, and parasitic DNA polymerization by reducing the availability of dNTP substrate for the enzyme.

Cellular dNTP concentrations are significantly varied among cell types [11]. Due to the close link between S phase-dependent dNTP biosynthesis and cellular DNA replication, dividing cells harbor an abundant amount of dNTPs compared to non-dividing cells [12]. Indeed, we previously reported that terminally differentiated/non-dividing monocyte-derived macrophages (MDMs), which are a HIV target cell type [13], have 22–320 times lower dNTP concentrations compared to actively dividing CD4^+^ T cells [13], [14]. Even though lentiviral reverse transcriptases (RT) have evolved to function at low dNTP concentrations, the limited dNTP availability contributes to a significant delay in proviral DNA synthesis in macrophages as compared to activated CD4^+^ T cells [13], [15]. However, some lentiviruses, such as HIV-2 and SIVsm, encode an accessory protein called viral protein X (Vpx) that overcomes the SAMHD1-induced dNTP depletion in non-dividing target cells [5], [7]. Upon infection, virally co-packaged Vpx promotes proteasomal degradation of SAMHD1 [16], [17], leading to the rapid elevation of cellular dNTP concentrations and ultimately the acceleration of proviral DNA synthesis [6], [8]. Both the Vpx-induced dNTP pool elevation and the promotion of viral reverse transcription were observed in several non-dividing viral target cell types which include macrophages [5], [6], [7], [8], DCs [18], [19] and resting CD4^+^ T cells [20], [21]. Moreover, all these cell types play a significant role in lentiviral pathogenesis. In addition, HIV-1 replicated more efficiently in monocytes isolated from Aicardi-Goutières Syndrome patients, who have mutations in *SAMHD1* [22]. The enhanced HIV-1 replication likely resulted from the elevated cellular dNTP pools due to loss of phosphohydrolase activity of mutated SAMHD1 [23]. A recent study also reported that other retroviruses such as feline immunodeficiency virus, bovine immunodeficiency virus, N-tropic and B-tropic murine leukemia viruses and equine infectious anemia virus were subject to restriction by SAMHD1 in macrophages, and this restriction was counteracted by the expression of Vpx [24]. These recent SAMHD1/Vpx studies support the hypothesis that SAMHD1 imposes a strong evolutionary selective pressure against lentiviral proviral DNA synthesis in non-dividing target cells by limiting dNTPs, the essential metabolic building blocks for DNA.

Interestingly, large double stranded DNA (dsDNA) viruses such as vaccinia virus, herpes simplex virus (HSV) and cytomegalovirus also infect non-dividing cells such as macrophages during the course of infection [25], [26], [27], [28], [29], [30]. However, unlike lentiviruses, these large dsDNA viruses encode dNTP biosynthesis proteins such as ribonucleotide reductase (RNR) and thymidine kinase (TK) that supply essential dNTP substrates for the viral DNA polymerase. Both of these genes are dispensable for HSV-1 viral replication in dividing cells, but are essential for replication under serum-starvation/non-dividing conditions where dNTP pools are limited [31], [32]. Thus, it is plausible that the dNTP biosynthesis machinery of dsDNA viruses promotes efficient replication in both dividing and non-dividing target cell types.

In this study we examined whether SAMHD1 affects the ability of vaccinia virus and HSV-1 to replicate in non-dividing cells. We observed that SAMHD1 controls the replication capacity of these dsDNA viruses by limiting the dNTP concentration.

## Results

### Vaccinia virus replication in primary human monocyte-derived macrophages was enhanced by Vpx-mediated degradation of SAMHD1

We investigated whether SAMHD1 affects the replication of vaccinia virus, the prototypical poxvirus, in primary human monocyte-derived macrophages (MDMs). Vaccinia is a large dsDNA virus that replicates entirely in the cytoplasm of the cell and has staged expression with early, intermediate, and late gene expression. Early genes are transcribed in the core of the virion upon entering the cytoplasm, whereas intermediate and late gene expression requires uncoating and replication of the dsDNA genome. For these studies, we utilized the Western Reserve (WR) strain of vaccinia virus that has the viral core protein (A4) fused to YFP (vA4-YFP), [33]. A4 is a late gene and is expressed after the viral genome has been replicated. It has been shown that the delivery of Vpx via lentiviral generated virus-like particles (VLPs) reduced the levels of SAMHD1, and increased the concentrations of dNTPs in MDMs [5], [6], [7], [8], [19], [21], [34], [35].

**Figure 1:**
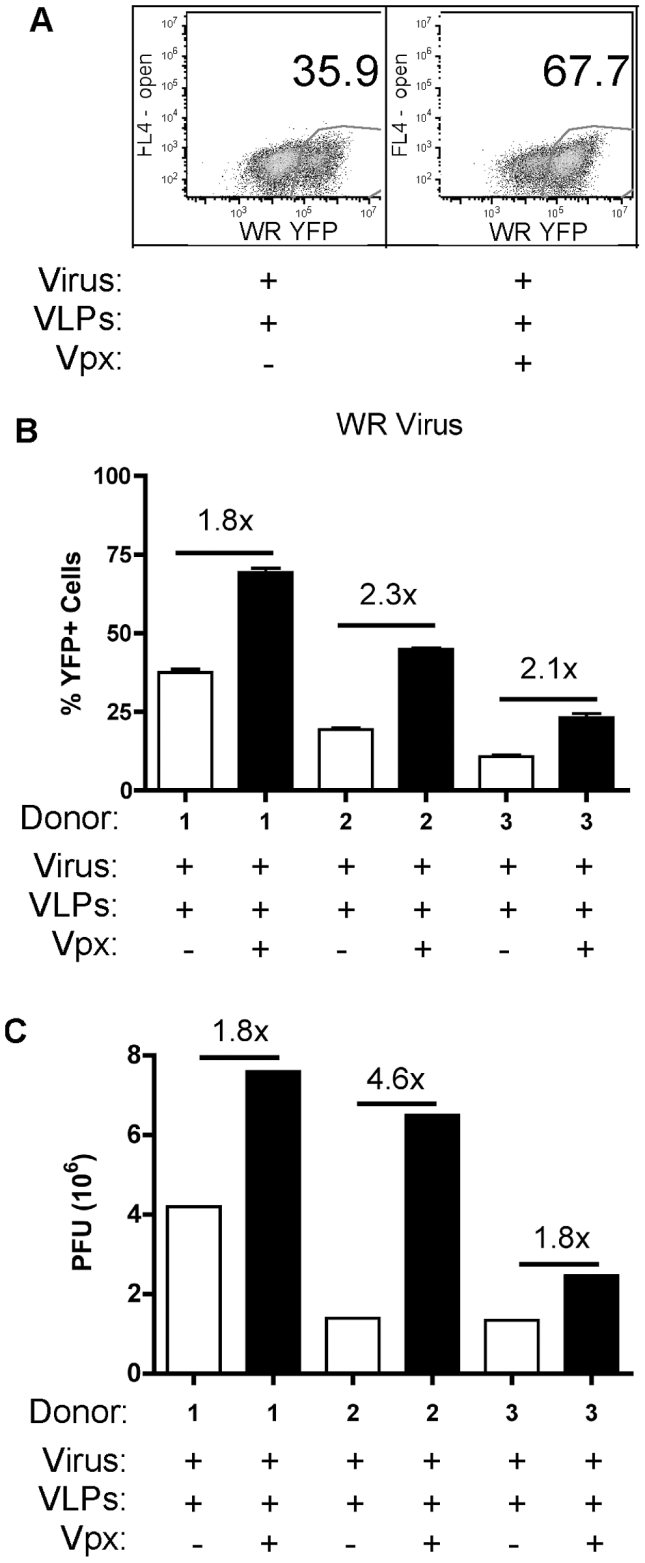
Vaccinia replication in MDMs. (A) Human primary monocyte-derived macrophages (MDMs) were pretreated with VLPs: Vpx+ and Vpx− for 24 h prior to infection with Western Reserve (WR) stain of vaccinia virus expressing the viral core protein A4 fused to YFP. A representative FACS dot blot shows the percent of infected MDMs at 24 hpi. (B) The means of three independent donors were graphed and are displayed as the percentages of YFP+ MDMs for the different VLPs treatment groups. (C) Plaque forming units (PFUs) were determined for three donors as described in Experimental Procedures. The fold changes between Vpx+ VLP and Vpx− VLP groups are displayed.

First, MDMs were pretreated with VLPs (containing or not containing Vpx) for 24 h, and then infected with virus. At 24 hpi, samples were collected and analyzed by various assays. As shown in Fig. 1A for a representative donor of MDMs, the productive infection frequency was monitored by examining the level of A4-YFP expression by flow cytometry. We then plotted the data for three independent MDM donors (Fig. 1B) and observed a measurable 1.8- to 2.3-fold increase in the frequency of YFP+ cells treated with Vpx+ VLPs as compared to the Vpx− VLP treated group (Fig. 1B). Six MDM donors were infected with A4-YFP WR virus, analyzed by flow cytometry for infection frequency and plotted in Fig. S1. The means (p<0.05; Mann-Whitney test) for the Vpx− versus Vpx+ VLP groups was 59% and 76%, respectively. Finally, we measured plaque-forming units (PFUs) for the different treatment groups, and found that Vpx+ VLP treatment led to a 1.8- to 4–6-fold increase in the amount of infectious viral particles produced as compared to Vpx− VLP treatment (Fig. 1C), To further confirm that the increases observed in infection frequency and PFUs were solely due to Vpx-mediated SAMHD1 degradation, a composite of all the experiments is presented in Fig. S2, showing that the no VLP treatment group was comparable to Vpx− VLP treatment group in all parameters.

**Figure 2:**
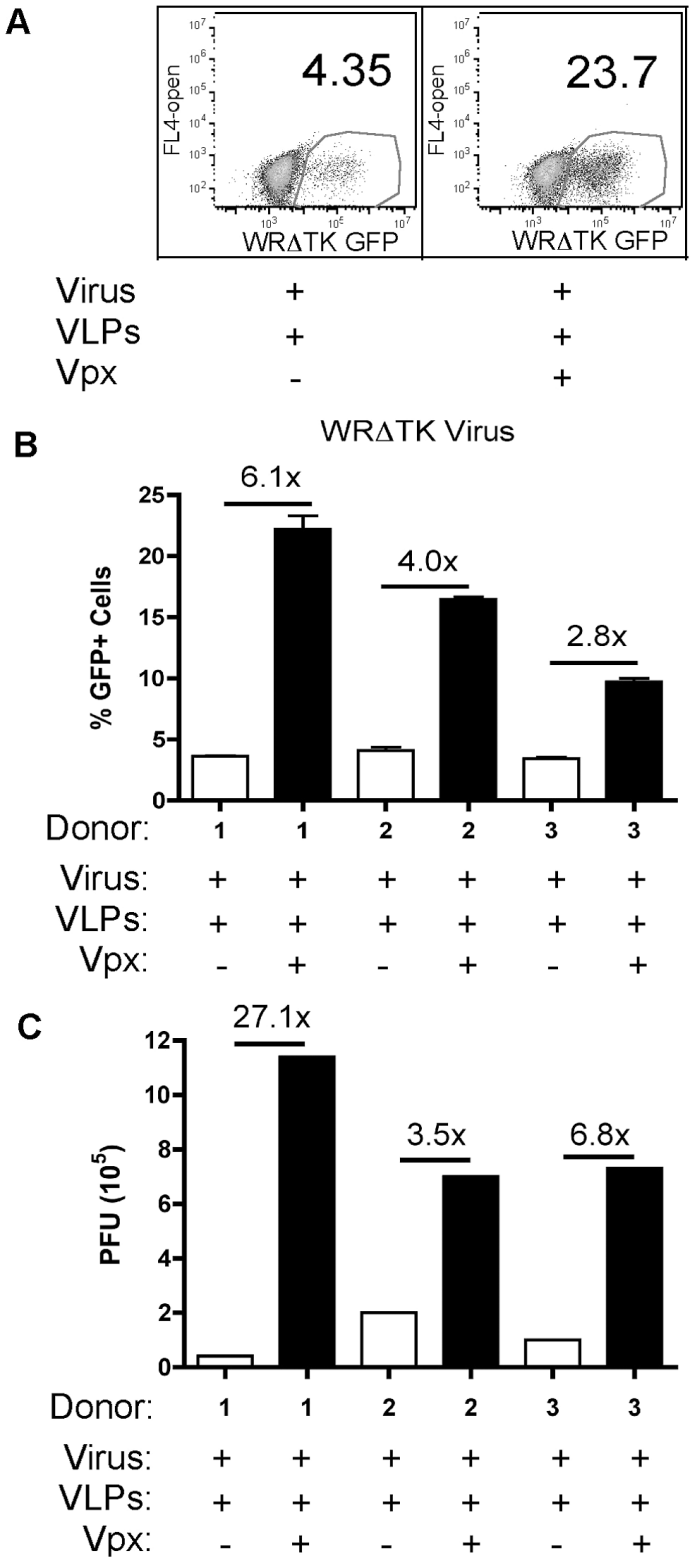
Analysis of WRΔTK replication in MDMs. (A) MDMs pretreated with Vpx+ or Vpx− VLPs for 24 h and then infected with a thymidine kinase deficient vaccinia virus expressing the late viral F13 protein fused to GFP (WRΔTK). (A) The percentage of GFP+ MDMs of three independent donors was determined using FACS, and the fold change between Vpx+ VLP and Vpx− VLP treatment was plotted. (B) The means of three independent donors were graphed showing the percentage of GFP+ MDMs for the different VLPs treatment groups. (C) PFUs were determined and plotted for the three donors infected with the WRΔTK virus. Fold changes were plotted comparing the Vpx+ VLP versus Vpx− VLP groups.

Vaccinia thymidine kinase (TK; J2R gene) is an early viral gene and is dispensable for viral replication in tissue culture; but it is important for virulence in animal models [36], [37]. TK is a central enzyme in the nucleotide salvage pathway and catalyzes the addition of a monophosphate to deoxythymidine (dT) [38]. To look at the involvement of viral TK in the infection of MDMs, the TK gene was removed and replaced with F13L-GFP fusion protein to generate, the WRΔTK (ΔJ2R) strain. The F13 gene is expressed late during infection and MDMs only become GFP+ when productively infected. To validate that the TK deletion, ΔJ2R, did not compromise viral growth, HeLa cells were pretreated with VLPs and then infected with WRΔTK (vΔJ2R/F13L-GFP) or parental WR (vA4-YFP) strain. We found that both strains infected HeLa cells comparably using an MOI of 0.5 PFU/cell, and did not require Vpx+ VLPs for enhancing infectivity (Fig. S3), which is consistent with published data showing that TK is not essential for infection in tissue culture [36]. To test the importance of viral TK, MDMs were pretreated with VLPs for 24 h and subsequently infected with WRΔTK virus. At 24 hpi, we quantified the frequency of GFP+ MDMs by flow cytometry (Fig. 2A, representative donor shown) and plotted the percentages of GFP+ MDMs for three independent donors (Fig. 2B). Similar to the parental WR strain, MDMs pretreated with Vpx+ VLP showed a 2.8- to 6.1-fold increase in the percentage of MDMs expressing the late protein F13L-GFP in the absence of the viral TK gene, indicating an enhancement of viral replication. Six MDM donors were infected with WRΔTK virus, analyzed by flow cytometry and plotted in Fig. S1. The mean percentages for viral infection were 9% and 24% (p<0.05) for the Vpx− VLP versus Vpx+ VLP groups, respectively. Finally, we observed 3.5- to 27.1-fold increase in PFUs between Vpx+ VLP versus Vpx− VLP treatments (Fig. 2C). PFUs were determined for differentiated THP-1 cells, which were treated with +/−Vpx VLPs and then infected with either WR or WRΔTK virus. THP1 cells showed similar trends to treatment as the primary human MDMs (Fig. S4). Collectively, data from experiments using WR and WRΔTK viruses suggest that Vpx-mediated SAMHD1 degradation enhances vaccinia virus infection in MDMs, with an even greater enhancement of infection in the absence of the viral encoded TK gene.

### Vaccinia virus infection does not promote SAMHD1 degradation in MDMs

**Figure 3:**
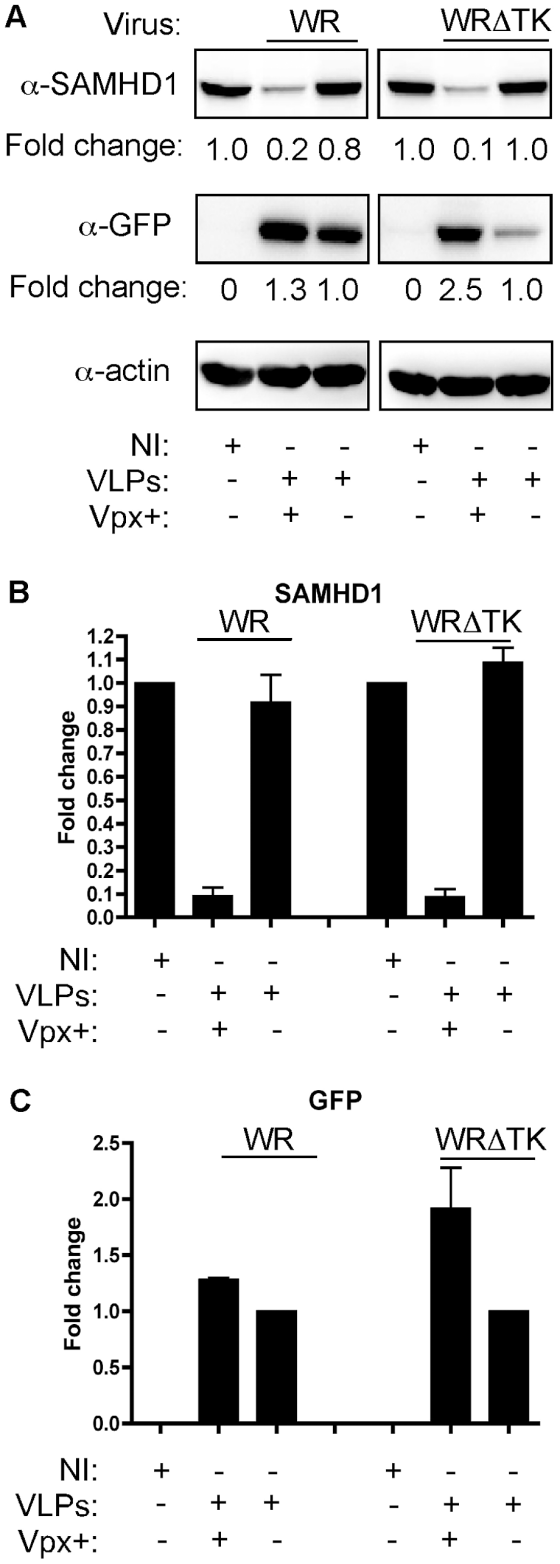
Expression of SAMHD1 in vaccinia-infected MDMs. (A) MDMs treated with Vpx+ or Vpx− VLP were infected (1 PFU/cell) with either WR or WRΔTK virus (NI: no infection). Cell lysates were generated 24 hpi and analyzed by Western blot using α-SAMHD1, α-actin, or α-GFP antibodies. (A) Representative immunoblots from one donor are shown. Immunoblots for SAMHD1 (B) and GFP (C) were quantified for three independent donors. Data were normalized to the amount of actin signal and were plotted for the amount of signal in the NI lane (set to 1.0) for SAMHD1 and the Vpx− VLP lane (set to 1.0) for GFP.

Several lentiviruses encode Vpx. It promotes the degradation of SAMHD1, leading to an increase in the dNTP pool and faster proviral DNA replication [10], [39], [40]. We tested whether vaccinia virus infection promoted degradation of SAMHD1 in MDMs. Cells were pretreated with Vpx+ or Vpx− VLPs for 24 h and were then infected with either WR or WRΔTK vaccinia viruses at 1 PFU/cell. The following day, MDMs were harvested and lysed to detect SAMHD1 levels by Western blot analysis. Vpx+ VLP treatment showed a substantial decrease in the amount of SAMHD1 (85–99% reduction in protein level as compared to uninfected MDM or infected Vpx− VLP treated MDMs (Fig. 3A; representative donor shown). SAMHD1 expression level data for the three donors are plotted in Fig. 3B. To monitor viral replication, cell lysates were incubated with a GFP antibody, which recognizes both A4-YFP and F13L-GFP (late gene protein products). Immunoblots showed that the Vpx+ VLP pretreated groups for either WR or WRΔTK virus had more protein detected than the Vpx− VLP pretreated groups (Fig. 3C), indicating more viral genome replication had occurred in the Vpx+ VLP treatment groups. These data show that vaccinia virus does not promote SAMHD1 degradation in MDMs, and that the enhanced viral replication capability in MDMs was mediated by Vpx+ VLP treatment.

### Vaccinia infection increases dNTP levels, which is further increased by Vpx+ VLP treatment

**Figure 4:**
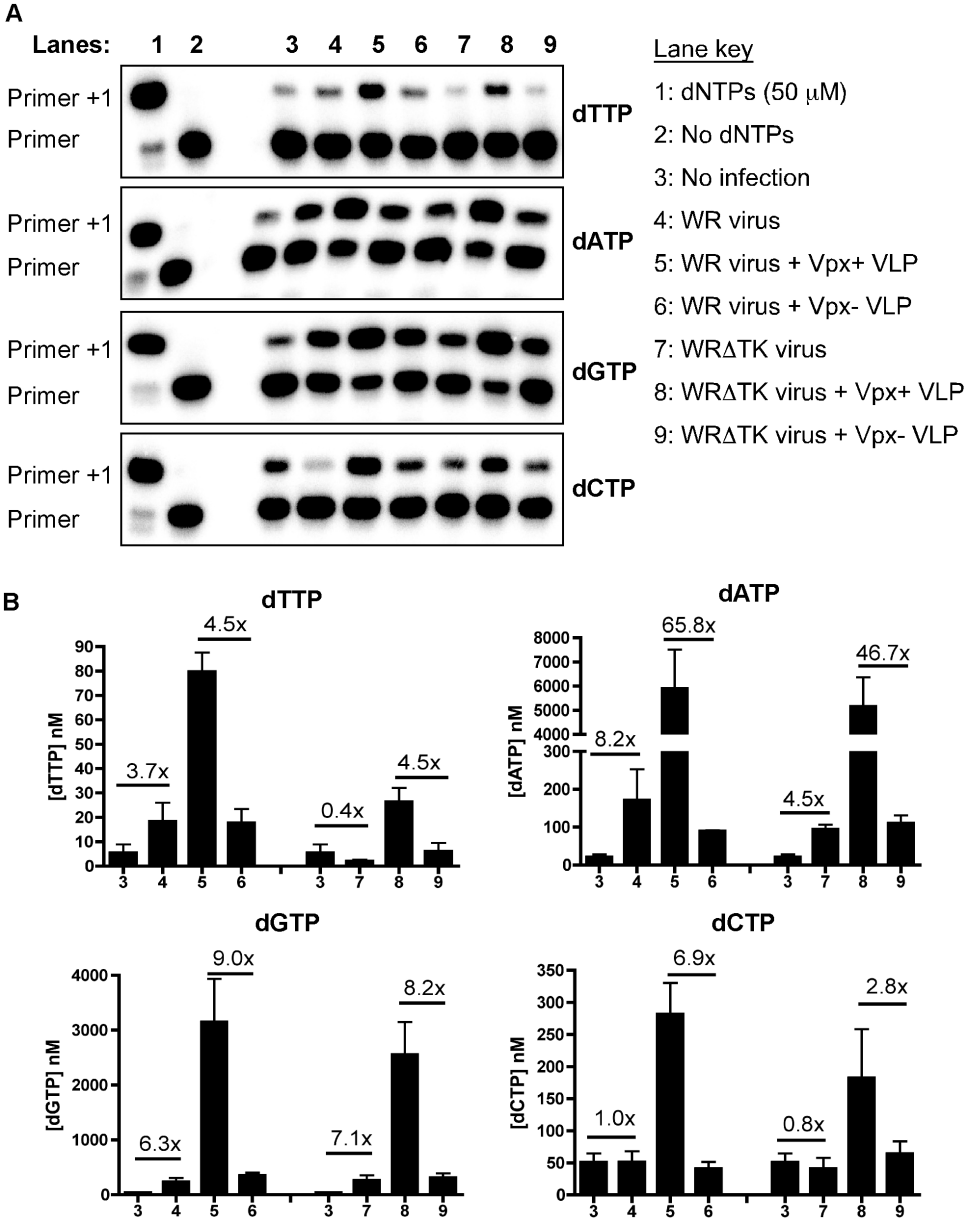
HIV-1 RT primer extension assay monitors dNTP levels. (A) MDM sample extracts were analyzed using a dNTP assay. 5′ ^32^P-end-labeled primer (“P”) was individually annealed to one of four different templates. The molar amount of the nucleotide extension product (“P+1”) is equal to that of each dNTP contained in the extracted samples, which allows us to calculate and compare the dNTP concentrations for the different treatments [13]. Representative gels are shown for one of three MDM donors monitoring the levels of dNTPs under various conditions. The lane key indicates the different treatment conditions used for each lane. (B) Quantitation of the raw data was plotted as bar graphs for each of the dNTP concentrations. Fold changes in dNTPs between different MDMs treatment groups were plotted.

Like other large dsDNA viruses, vaccinia virus encodes several dNTP biosynthesis proteins such as TK and RNR [41], [42]. Therefore, we examined cellular dNTP concentrations in MDMs infected with vaccinia virus in the presence and absence of Vpx treatment. To do this, we used a highly sensitive HIV-1 RT-based primer extension assay [13] to monitor changes in dNTP levels for each of the four dNTPs. In this assay (Fig. 4), the level of the extended primer product (Primer +1) is indicative of the dNTP level. Both fold increases and absolute dNTP concentrations for dATP, dGTP, dCTP and dTTP were determined for the various treatment groups (Fig. 4B). As shown in lane 3 of Fig. 4A, uninfected MDMs displayed very low levels of dNTPs, indicative of the low cellular dNTP level in MDMs. MDMs infected with WR (Fig. 4B, lane 4) showed elevated levels for dATP (8.2-fold), dGTP (6.3-fold) and dTTP (3.7-fold), while dCTP (1.0-fold) remained unchanged. Our results demonstrate that virus encoded dNTP biosynthesis machinery was able to elevate the cellular dNTP levels in MDMs. However, when we analyzed the dNTP samples extracted from MDMs infected with WRΔTK virus only dGTP (7.1-fold) and dATP (4.5-fold) were elevated while dCTP (0.8-fold) and dTTP (0.4-fold) were reduced as compared to uninfected MDMs. This demonstrates that WRΔTK is deficient in TK activity. These data indicate that the increased replication of WR strain as compared to WRΔTK strain in MDMs is the result, in part, of increased dTTP biosynthesis.

When MDMs were pretreated with Vpx+ VLP and were infected with either WR or WRΔTK viruses, all four dNTPs increased (Fig. 4B, lane 5 compared to lane 6 for WR, and lane 8 compared to lane 9 for WRΔTK). Comparing Vpx+ VLP versus Vpx- VLP treated MDMs, dTTP was increased by 4.5-fold; dATP was increased by 65.8-fold and 46.7-fold; dGTP was increased by 9.0-fold and 8.2-fold; dCTP had increased by 6.9-fold and 2.8-fold for WR and WRΔTK viruses, respectively. Collectively, vaccinia infection of MDMs alone only modestly elevated the cellular dNTP pools, which was further enhanced by Vpx+ VLP treatment. However, the measured dNTP pool levels for vaccinia-infected MDMs may be underestimated, since the viral DNA polymerase utilizes dNTPs in order to replicate its genome.

### SAMHD1 also controls infection of HSV-1 viral replication in THP-1 cells

**Figure 5:**
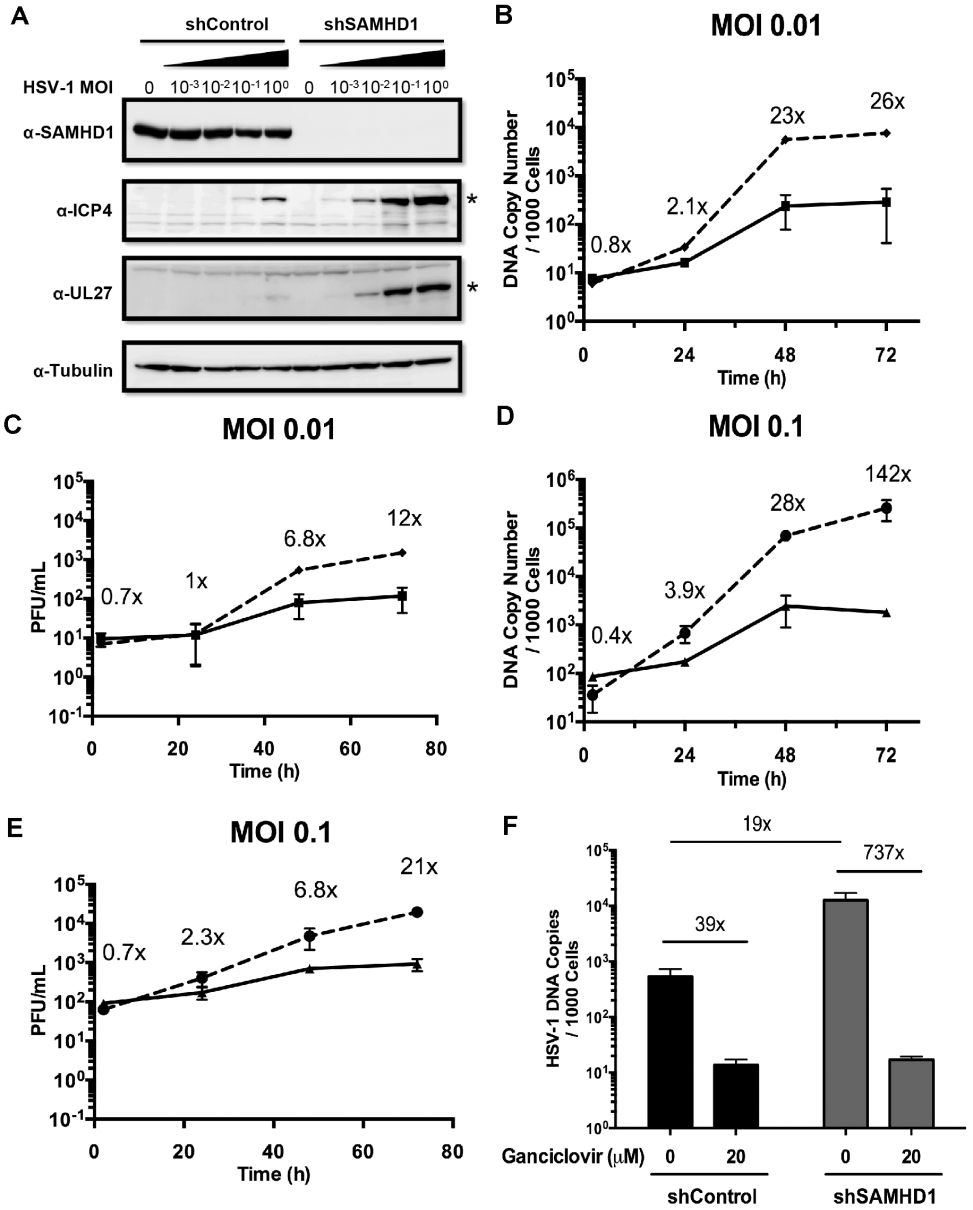
SAMHD1 inhibits HSV replication. (A) Western blot analysis of lysates obtained from PMA-stimulated THP-1 shControl and shSAMHD1 cells, infected with varying MOIs of HSV-1 KOS, was performed to determine the levels of SAMHD1, the HSV-1 viral proteins UL-27 and ICP4 (denoted by asterisks), and a tubulin loading control. (B) Real-time PCR was carried out to determine HSV-1 DNA levels after HSV-1 KOS infection in PMA-stimulated shControl (solid line) and shSAMHD1 (dashed line) THP-1 cells at an MOIs of 0.01 over 72 h. (C) PFUs were measured for THP-1 cells infected at an MOI of 0.01. (D) Real-time PCR measured HSV-1 DNA levels for THP-1 cells infected at an MOI of 0.1. (E) PFUs were measured for THP-1 cells infected at an MOI of 0.1. (F) HSV-1 DNA levels were determined after HSV-1 infection of PMA-stimulated shControl and shSAMHD1 THP-1 cells in the presence or absence of 20 µM ganciclovir. The results are representative of two independent experiments performed in duplicate.

Next we validated that SAMHD1 controls replication of another large dsDNA virus, HSV-1. For these studies, we infected a human monocytic cell line, THP-1 cells, with an HSV-1 strain (HSV-1 KOS). THP1 cells undergo differentiation to a macrophage-like cell by Phorbol 12-myristate 13-acetate (PMA) treatment. In addition, we also used PMA-treated THP-1 cell lines that stably express SAMHD1-specific shRNA (shSAMHD1) or control shRNA (shControl). As previously reported [34], we confirmed knockdown of SAMHD1 in the differentiated THP-1 cells expressing SAMHD1 specific shRNA with HSV-1 infections at various MOIs, but not in cells expressing the control shRNA (MOI “0” in Fig. 5A). In addition, the SAMHD1 level remained unchanged even with a MOI of 1 for HSV-1 KOS in the shControl THP-1 cells, indicating that like vaccinia virus, HSV-1 does not down-regulate SAMHD1 expression. Next, we monitored the expression of HSV-1 intermediate-early viral protein ICP-4 and late viral protein UL-27 by Western blot analysis. Higher levels of ICP4 and UL-27 were observed in the shSAMHD1 cells as compared with shControl cells, with the most pronounced differences detected at MOIs of 0.1 and 1, (*, Fig. 5A), implying that SAMHD1 suppresses HSV-1 KOS in differentiated THP-1 cells. Next, shControl and shSAMHD1 THP-1 cells were infected with WT HSV-1 KOS virus at MOIs of 0.01 and 0.1, and viral DNA replication was monitored by real-time PCR to determine the level of initial viral input (“2 h” time point) (Fig. 5B & D) and also my measuring PFUs (Fig. 5C & E). By 72 hpi, the HSV-1 copy number in shSAMHD1 cells was 26-fold (Fig. 5B; MOI 0.01) and 142-fold (Fig. 5C; MOI 0.1) higher than in the corresponding shControl cells. PFUs increased by 12-fold and 21-fold at 72 hpi for the different groups infected with an MOI 0.01 and 0.1, respectively. Indeed, both HSV-1 genome replication and viral production was clearly augmented in shSAMHD1 cells (dashed lines) as compared with shControl cells (solid lines).

To further validate that an increase in viral production occurred, differentiated THP-1 cells were pretreated with ganciclovir, a nucleoside analogue that blocks HSV-1 DNA replication. As shown in Fig. 5F, we detected inhibition of HSV-1 DNA replication by real-time PCR in both shControl and shSAMHD1 THP-1 cells pretreated with 20 µM ganciclovir, which is sufficient to inhibit viral replication in dividing cells [43]. However, in the absence of drug, we observed a 39-fold increase in DNA replication in the shControl cells and a 737-fold increase in the shSAMHD1 THP-1 cells (Fig. 5D). Without ganciclovir treatment, a 19-fold difference was observed between shControl cells versus shSAMHD1 THP-1 cells. These data support the notion that SAMHD1 controls the DNA replication of HSV-1 KOS in differentiated THP-1 macrophages.

### SAMHD1 inhibition of HSV-1 infection requires differentiation of THP-1 cells

**Figure 6:**
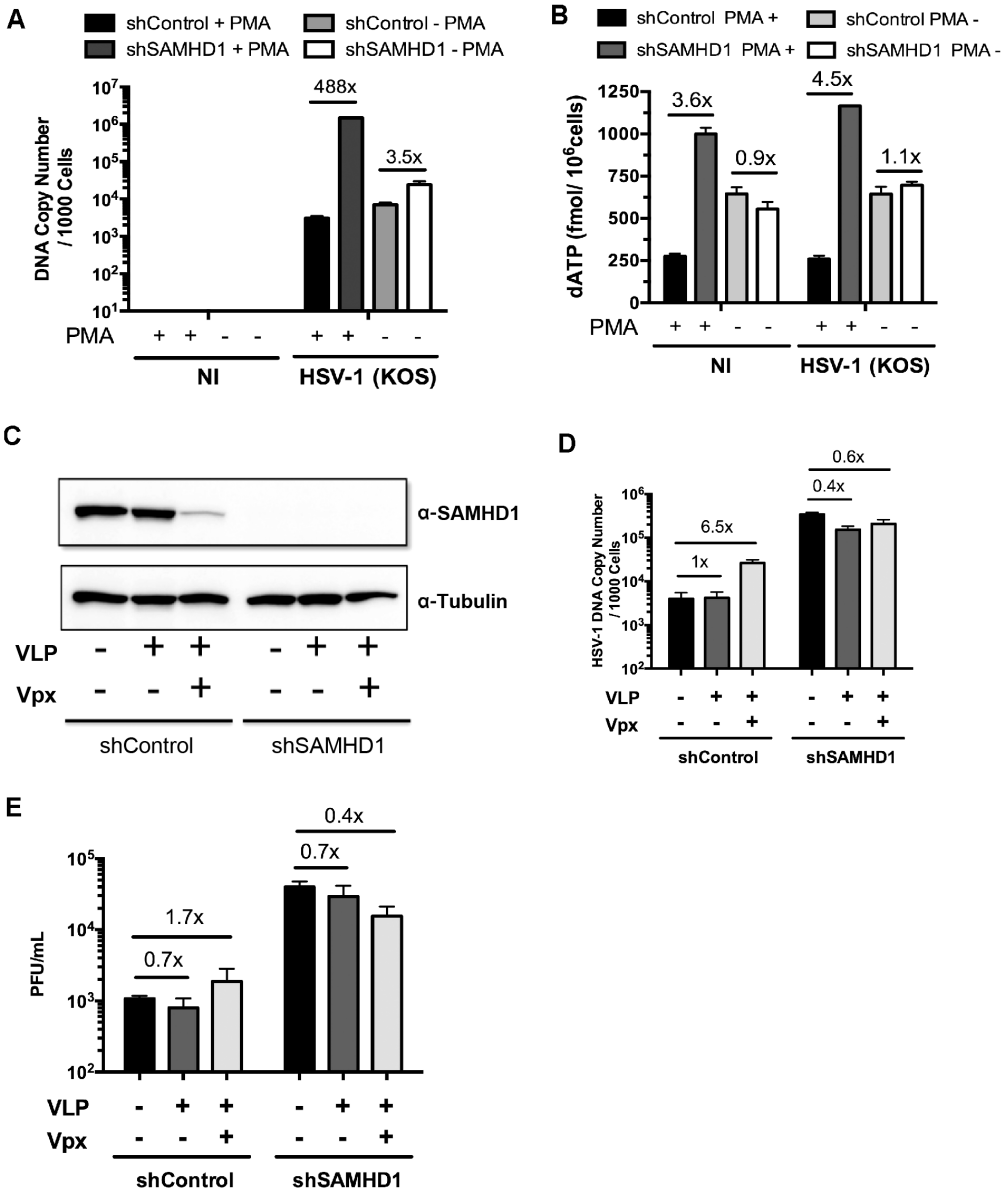
Vpx-mediated down regulation of SAMHD1 augments HSV-1 KOS infection of THP-1 cells. (A and B) shControl and shSAMHD1 THP-1 cells were infected by HSV-1 (KOS) at a MOI of 0.1 under non-dividing or dividing conditions for 48 h. These results are representative data of three independent experiments performed in duplicate. (A) HSV-1 DNA copy numbers were analyzed by real-time PCR and (B) the level of dATP in the cells was determined by the HIV-1 RT primer extension assay as shown in Fig. 4. (C) PMA-stimulated shControl and shSAMHD1 THP-1 cells were inoculated with medium containing vehicle, Vpx- VLP, or Vpx+ VLP for 24 h and infected with HSV-1 WT virus at a MOI of 0.1. Cell lysates were generated 48 hpi and protein levels of SAMHD1 and tubulin were determined by western blot analysis. The results are representative of three independent experiments performed in duplicate. (D) Real-time PCR was used to monitor HSV-1 DNA levels. (E) PFUs for shControl and shSAMHD1 THP-1 cells were measured for the different VLP treatment groups. Fold changes between the different groups were determined and displayed.

Next, we tested whether the SAMHD1-mediated suppression of HSV-1 KOS requires the differentiation of the THP-1 cells. For this test, THP-1 cells were infected with HSV-1 KOS at a MOI of 0.1 for 48 h in the presence of PMA (maturated, non-dividing cells) or in the absence of PMA (dividing cells). We found that the copy number of HSV-1 DNA was higher in PMA differentiated shSAMHD1 cells than in shControl cells by 488-fold (Fig. 6A). In contrast, without differentiation, only a 3.5-fold increase in HSV-1 KOS DNA replication was observed in shSAMHD1 cells as compared to shControl cells (Fig. 6A). These data suggest that SAMHD1-mediated inhibition of HSV-1 infection is dependent on the differentiation stage of the cells.

Next, we examined dATP concentration in differentiated and undifferentiated THP-1 cells that were either infected or uninfected with HSV-1 KOS virus. As shown in Fig. 6B, both the uninfected and infected differentiated shSAMHD1 cells showed elevated dATP pools (3.6-fold and 4.5-fold, respectively) as compared to the shControl THP-1 cells, suggesting that HSV-1 infection alone did not promote a significant change in cellular dATP levels. Between undifferentiated shControl and shSAMHD1 THP-1 cells, no difference in dATP concentration was observed for either NI (0.9-fold) or infected (1.1-fold) groups. These data suggest that the dNTP depletion by SAMHD1 can negatively regulate the dNTP pool size even in the presence of HSV-1 dNTP biosynthesis in differentiated THP-1 cells.

### Vpx-dependent degradation of SAMHD1 augments HSV-1 infection in PMA-differentiated THP-1 cells

Next we tested whether Vpx treatment could enhance HSV-1 replication in differentiated THP-1 cells. First, the shControl and shSAMHD1 THP-1 cells were treated with Vpx− or Vpx+ VLPs, and the SAMHD1 level was monitored by Western blot analysis. As shown in Fig. 6C, Vpx+ VLP treatment effectively degraded SAMHD1 in the shControl THP-1 cells, while shSAMHD1 THP-1 cells showed no detectable SAMHD1 expression regardless of Vpx+ VLP treatment. Using real-time PCR, we found that Vpx+ VLP treatment elevated HSV-1 replication by 6.5-fold for differentiated shControl THP-1 cells (Fig. 6D), while Vpx- VLP treatment was comparable to no VLP treatment (1-fold). No change in viral DNA copy number was detected after Vpx+ VLP treatment in the differentiated shSAMHD1 THP-1 cells (Fig. 6D), which is likely due to nearly complete inhibition of SAMHD1 by the shRNA. We observed a 1.7-fold increase in PFUs for the Vpx+ VLP treated shControl THP-1 cells as compared to NI control cells (Fig. 6D) and a slight decrease in PFUs for VLP treatment shSAMHD1 groups. Collectively, these data show that Vpx+ VLP treatment can enhance both the viral genome copy number and PFUs in differentiated shControl THP-1 cells, while have no significant increase in these two parameters in the differentiated shSAMHD1 THP-1 cells, which already have greatly diminished SAMHD1 protein level.

### Vpx promotes HSV-1 replication in primary human DCs and MDMs

**Figure 7:**
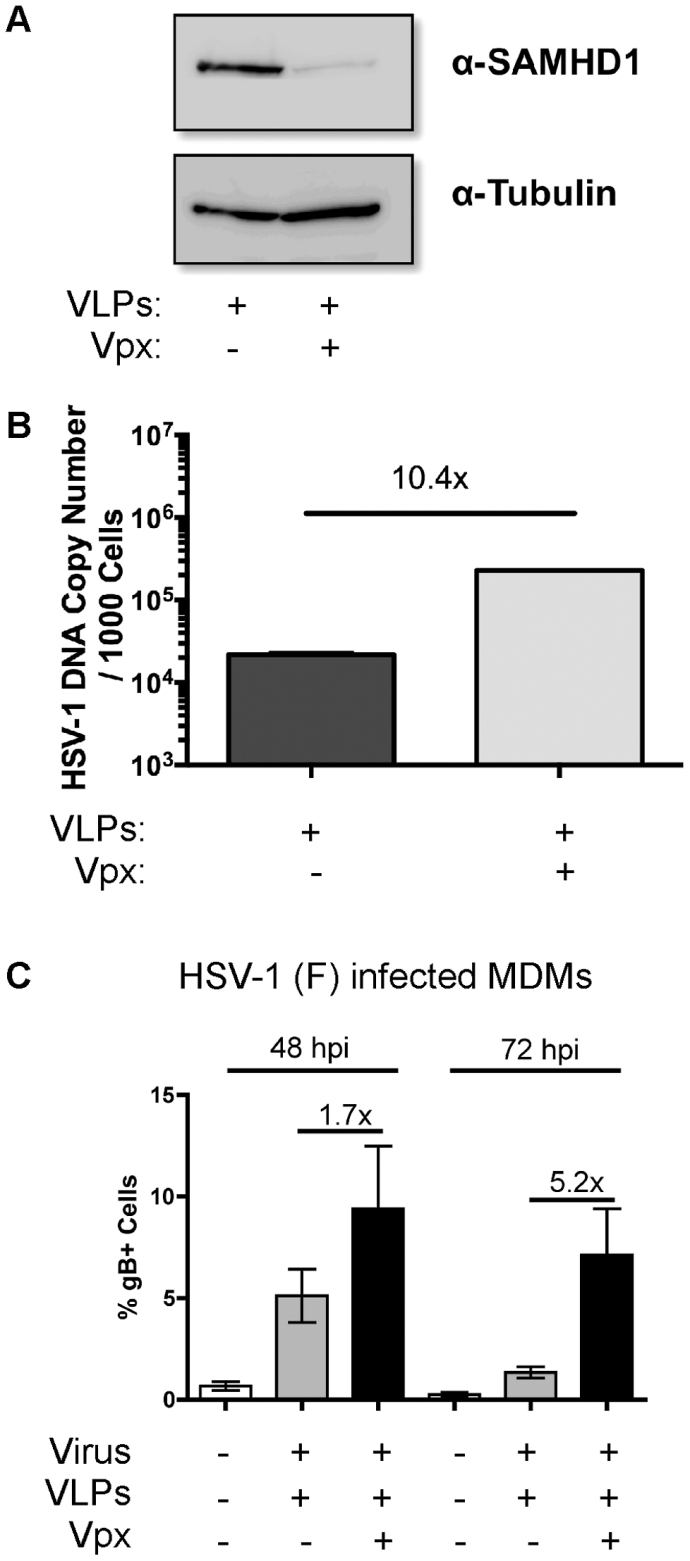
SAMHD1 augments HSV-1 infection in human primary DCs and MDMs. Primary mature DCs were inoculated with medium containing vehicle, Vpx− VLP, or Vpx+ VLP for 48 h and then infected with HSV-1 KOS at a MOI of 0.1. Cell lysates were generated at 48 hpi and (A) western blot analysis for SAMHD1 and tubulin were performed. (B) Real-time PCR for HSV-1 DNA levels were determined 48 hpi. (C) Primary human MDMs were pretreated with VLPs before infecting with HSV-1 F strain (0.5 PFU/cell). Infection was monitored for intracellular staining of viral gB protein by FACS analysis and the data graphed as a percentage of gB positive MDMs.

HSV-1 also productively replicates in primary DCs, which are non-dividing cells [44], [45]. We recently reported that Vpx enhances HIV-1 replication in DCs by targeting SAMHD1 for degradation [18]. Thus, we investigated whether the degradation of SAMHD1 also augments HSV-1 infection in human primary DCs. Cells were infected with HSV-1 KOS after the addition of Vpx− or Vpx+ VLPs. Similarly to PMA-differentiated THP-1 cells, the addition of Vpx+ VLPs caused a decrease in SAMHD1 protein levels (Fig. 7A), and enhanced HSV-1 KOS infection by 10.4-fold as compared to primary DCs treated with Vpx− VLPs (Fig. 7B).

Finally, in order to generalize the interplay between SAMHD1 and HSV-1 in macrophages, we employed another HSV-1 strain, HSV-1 F strain [46] and conducted studies using MDMs infected at 0.5 PFU/cell. Vpx+ VLP treatment of MDMs demonstrated 1.7-fold and 5.2-fold enhancement at 48 and 72 hpi, respectively, for HSV-1 (F) infection by intracellular straining of the viral gB protein (Fig. 7C). Collectively, these data show that SAMHD1 suppresses HSV-1 replication in primary human DCs and MDMs, and that SAMHD1 degradation promotes HSV-1 replication in these two mature myeloid cell types.

## Discussion

Several studies have shown the necessity of both innate and adaptive immune responses in order to control both herpes and poxvirus infections [47], [48], [49], [50], [51]. Macrophages are part of the innate immune response and are rapidly recruited to sites of infection. It is therefore not surprising that these cells have developed strategies to limit pathogen replication within them. A series of recent studies highlighted SAMHD1, a cellular dNTP triphosphohydrolase, as an anti-lentiviral restriction factor predominantly found in myeloid cells [5], [6], [7], [8], [24], [40], [52]. Since dNTPs are universal substrates for cellular, parasitic, and viral DNA polymerases, it seems likely that cellular dNTP concentrations could influence the genome replication of all dsDNA viruses. We attempted to expand the role of SAMHD1 as a restriction factor against large dsDNA viruses such as vaccinia virus and HSV-1. Indeed, the results presented in this study support the theory that SAMHD1 suppresses the replication capacity of these dsDNA viruses in a similar manner observed with HIV-1 and HIV-2 lentiviruses, by depleting the cellular dNTP pools and thus limiting the substrate availability for viral DNA polymerases.

Viral replication occurs in different compartments for vaccinia virus and HSV-1. Like retroviruses, vaccinia virus undergoes DNA synthesis outside the nucleus and sets up a cytoplasmic viral factory for replication of its genomic DNA [53]. In contrast, the HSV-1 genome is replicated in the host cell nucleus [54] and is dependent on nuclear import [55]. This difference in cellular localization of viral genome replication may contribute to the complexity in understanding each virus; but clearly from our data, high cellular dNTP pools appears to be critical for efficient viral genome replication regardless of the cellular compartment in which it takes place in. It is surprising that neither vaccinia virus nor HSV has evolved to target SAMHD1 for degradation. Since cellular nucleotides are dispersed throughout the cell [12], SAMHD1 can deplete dNTPs and restrict viral replication of DNA viruses regardless of their replication site within a cell [56].

HSV-1 and vaccinia virus have been reported to hamper adaptive immunity upon infection of myeloid cells. HSV-1 infection of DCs promotes the down regulation of CD83 [45], a co-stimulatory molecule. This in turn may limit T cell activation against this pathogen. Vaccinia virus infection inhibits MHC class II presentation on the surface of macrophages [57] and DCs [58]. Therefore, the antiviral role of SAMHD1 in myeloid cells may be to limit viral replication, in an environment of decreased antigen-presentation by myeloid cells.

Interestingly, unlike retroviruses, these large dsDNA viruses are equipped with their own dNTP biosynthesis machinery such as TK and RNR, to aid in genome replication when dNTP concentrations are suboptimal. However, TKs from vaccinia and HSV-1 are most similar in sequence and structure to human TK1 (hTK1) [59]. hTK1 is also cell cycle regulated [60], being degraded in G0 cells, such as MDMs. Moreover, the activity of human RNR is coupled to DNA replication [61]. Collectively, MDMs have very low dNTPs, making them very restrictive to infection. Indeed, infection with vaccinia virus alone promoted a measurable increase in dTTP, dATP, and dGTP concentrations in MDMs (Fig. 4). With the exception of dTTP, WRΔTK virus also induced a similar increase, albeit not to the same levels. The increase in dNTPs was not enough to promote a robust infection of MDMs by vaccinia virus. Indeed, the antiviral restriction potential of SAMHD1 became greater when using WRΔTK (vΔJ2R/F13L-GFP) virus to infect MDMs. This observation proposes that dsDNA viruses may have evolved to encode their own viral dNTP biosynthesis enzymes to change the dNTP pool ratios in their favor, since vaccinia contains a high 66.6% AT rich genome [62], whereas HSV-1 contains a 68% GC rich genome [63]. However, both viruses have not adapted to escape from the replicative restriction pressure induced by SAMHD1 in macrophages and DCs, since they do not degrade SAMHD1 (Fig. 3A and 5A). Collectively, our findings validate that SAMHD1 acts as a host restriction factor against large dsDNA viruses in non-dividing target cells.

## Materials and Methods

### Ethics statement

For vaccinia virus and HSV-1 F strain experiments, primary human primary monocytes were obtained from human buffy coats (New York Blood Services, Long Island, NY). These are pre-existing materials that are publicly available, and there is no subject-identifying information associated with the cells. As such, the use of these samples does not represent human subjects research because: 1) materials were not collected specifically for this study, and 2) we are not able to identify the subjects. For HSV-1 KOS experiments, monocytes were obtained from the buffy coats of healthy volunteers with the approval of the Kyoto University ethics committee. Written informed consent forms were obtained from all participants.

### Cells

Primary human monocytes were isolated from the peripheral blood buffy coats by positive selection using MACS CD14+ beads as previously described [64] or by a Dynabeads Untouched Human Monocytes kit according to the manufacturer's protocol. For macrophage differentiation, monocytes were matured for seven days in RPMI medium containing 10% FCS, Pen/Strep antibiotics and 5 ng/ml human recombinant GM-CSF (R&D Systems) before use in experiments. For dendritic cell differentiation, monocytes were matured for 5 days in RPMI medium containing 10% FCS, Pen/Strep antibiotics, 100 ng/mL human recombinant GM-CSF, and 100 ng/mL human recombinant IL-4 (R&D Systems). THP-1 cells containing shControl and shSAMHD1 were kindly provided by Dr. Nathaniel R. Landau (New York University School of Medicine). THP-1 cell lines were maintained in RPMI medium with 10% FCS under 0.5 µg/mL puromycin selection before maturation with 50 nM PMA. HeLa cells were maintained with RPMI medium containing 10% FCS and Pen/Strep antibiotics. Vero cells were maintained with DMEM medium containing 10% FCS and Pen/Strep antibiotics and used to determine the PFU for the HSV-1 KOS virus. BSC-40 cells were maintained as previously described and used to determine PFU for WR (vYFP-A4) and WRΔTK (vΔJ2R/F13L-GFP) vaccinia viruses [33].

### Viruses and infection

Vaccinia virus (VV) vYFP-A4 and vTF7.3 were kind gifts of Bernard Moss. To construct the VV WRTK- reporter virus, the F13L gene was replaced with the coding sequence of F13L-GFP in the recombinant VV vTF7.3 using a strategy that has already been described [65]. The resulting recombinant vaccinia virus, vΔJ2R/F13L-GFP, has the TK gene (J2R) removed and expresses the late protein F13L fused to GFP. HSV-1 KOS was kindly provided by Dr. Sandra Weller (University of Connecticut Health Center). Wild type HSV-1 F strain was kindly provided by Dr. Bernard Roizman (University of Chicago; [46]). For HSV-1 KOS infection experiments, THP-1 cells were stimulated with 50 ng/mL of PMA overnight, washed on day two with PBS, and replaced with fresh medium. On the third day, HSV-1 KOS adsorption was carried out for one hour at 37°C at the indicated MOI, cells were washed twice with PBS, replaced with fresh media and infection was allowed to continue for the indicated time points. Unless indicated otherwise, a MOI 0.1 was used for all HSV-1 experiments.

### VLP generation

Six T225 flasks containing 293T cells were transfected with 40 µg of pVpx− VLP or pVpx+ VLP (kindly provided by Drs. Florence Margottin-Goguet and Nathaniel Landau) and 20 µg of pVSVg at a ratio of 1 µg of DNA to 3 µl of polyethylenimine (1 mg/ml). The following day, medium were discarded and replaced with fresh DMEM medium (5% FBS and antibiotics). On days 2–3 after transfection, the medium was collected and replaced with fresh medium. On the day of collection, medium was centrifuged at 1200 rpm for 5 min to remove cells. Supernatant was subsequently filtered through a 0.45-µm membrane (Corning Inc.). Supernatant was overlaid on top of 5 ml of a 25% sucrose cushion (25% (w/v) sucrose, 10 mM Tris-HCl [pH 7.5], 0.1 M NaCl and 1 mM EDTA). VLPs were concentrated at 28,000 rpm for 90 min by ultracentrifugation. Supernatant was aspirated, and pellets were suspended in 600 µl of serum-free DMEM. Supernatant was centrifuged for 1 min at 14K rpm to remove debris. Aliquots (50 µl) were stored at −80°C. The p27 antigen level was determined using an ELISA kit (Advanced BioScience Laboratories, Inc., Rockville MD). A minimum of 145 ng of p27/million cells was used.

### Viral plaque assay

The procedure for the vaccinia plaque assay used has been described previously [33]. Briefly, confluent monolayers of BSC-40 cells were infected with the indicated viruses. At 2 hpi, the inoculum was removed and cell monolayers were overlaid with semisolid medium. Three days after infection, cell monolayers were stained with crystal violet and imaged. For the HSV-1 plaque assay, supernatants obtained from HSV-1-infected THP-1 cells were serially diluted and then inoculated onto Vero cells seeded on a 6-well polystyrene plate. After a one hour adsorption at 37°C, cells were replaced with fresh medium containing the heavy chain of immunoglobulin G. After 3 dpi, the cells were fixed with 100% methanol for 5 min at room temperature and then stained with crystal violet solution (20% ethanol and 1 mg/ml of crystal violet) for 20 min at room temperature followed by washing with distilled water to remove the staining solution. Visible plaques were quantified.

### Primer extension assay

MDMs were lysed with 60% cold methanol. Cellular debris was cleared by 14K rpm centrifugation. Supernatant was dried using a speedvac. Pellets were resuspended in 20 µl water. Two microliters of sample were used in the primer extension assay. 5′ ^32^P-end-labeled primer (“P”; 5′-GTCCCTCTTCGGGCGCCA-3′) was individually annealed to one of four different templates (3′-CAGGGAGAAGCCCGCGGTN-5′), and this template∶primer complex was extended by HIV-1 reverse transcriptase generating one additional nucleotide extension product (“P+1”) for one of four dNTPs contained in the dNTP samples extracted form the cells. In this assay, the molar amount of the P+1 product is equal to that of each dNTP contained in the extracted samples, which allows us to calculate and compare the dNTP concentrations for the different treatments [13].

### Western blot analysis

Samples were processed in RIPA buffer containing 1 µM DTT, 10 µM PMSF, 10 µl/ml phosphatase inhibitor (Sigma) and 10 µl/ml protease inhibitor (Sigma). The cells were sonicated with 3×, 5 second pulses, to ensure compete lysis. Cellular debris was removed by 15K rpm centrifugation for 10 min. Supernatants were stored at −80°C before use. Cell lysates were resolved on 4–12% Bis-Tris NuPAGE gels (Invitrogen) and were transferred to nitrocellulose membranes and detected as described in the Figure legends using the following antibodies: mouse anti-GFP mAb (Roche), mouse anti-SAMHD1 mAb (Abcam), mouse anti-SAMHD1 mAb (kindly provided by Dr. Oliver Schwartz, [52]), mouse anti-tubulin mAb (Sigma), mouse anti-HSV-1 ICP4 mAb (Virusys), anti-UL27 and anti-ß-actin mouse mAb. HRP and Cy-5 conjugated, anti-mouse and anti-rabbit secondary antibodies were purchased from Jackson ImmunoResearch Laboratories and Cell Signaling. HRP was detected using chemiluminescent reagents (Pierce) following the manufacturers instructions. The fluorescent and chemiluminescent signals were captured using a Kodak Image Station 4000 mmPro (Carestream Health), outfitted with appropriate filters, and a Fujifilm LAS4000. Blot was striped and re-probed for actin. Images were captured using BioRad ChemiDoc Imager.

### FACS analysis

Samples were fixed with 4% formaldehyde and data collected using an Accuri C6 flow cytometer to monitor GFP/YFP expression at 24 h post infection. MDMs infected with HSV-1 (F) were fixed for 20 min at RT and then stained with primary anti-gB antibody (ab6506). Cells were washed and stained with secondary goat anti-mouse-PE (Southern BioTech). Samples were collected using Accuri C6 flow cytometer. FCS data files were analyzed using FlowJo software (TreeStar).

### Real-time PCR

Nucleic acids were extracted by the urea lysis method as previously described [66]. For HSV-1 DNA quantitation, 100 ng of nucleic acids was used for real-time PCR and the HSV-1 genome copy number was calculated based on a standard curve generated using a plasmid containing the UL27 gene. Thermocycler conditions for the real-time PCR were as follows: 95°C for 10 min and 95°C for 15 s plus 60°C for 1 min for 40 cycles. The primers used for real-time PCR are as follows: HSV-1 UL27 Forward (5′-TCGCCTTTCGCTACGTCAT-3′), HSV-1 UL27 Reverse (5′-GGTTCTTGAGCTCCTTGGTGG-3′), GAPDH Forward (5′-GCAAATTCCATGGCACCGT-3′), and GAPDH Reverse (5′-TCGCCCCACTTGATTTTGG-3′).

### Graphing and statistical analysis

Prism software was used for plotting the data. All the data sets greater than three samples were first compared using ANOVA for significant differences with the means. Post-analysis was the done using Mann-Whitney test to determine significant difference between two groups.

## Supporting Information

Figure S1 **Infection of MDMs donors with different viruses.** A total of six MDMs donors were analyzed for the ability of Vpx VLP to enhance the infection of WR and WRΔTK strains. Mann-Whitney test was performed and significant differences indicated with * and p<0.05.
(TIFF)

Figure S2 **Direct comparison of WR and WRΔTK viruses for one donor.** FACS data were plotted for the percentage of (A) WR (YFP+) and (B) WRΔTK (GFP+) cells. PFU for (C) WR and (D) WRΔTK infected MDMs. The non-infected (NI) MDMs had zero PFUs detected. (E) Immunoblot analysis for SAMHD1 expression at 48 h after VLP treatment and 24 hpi. The NI MDMs had comparable SAMHD1 expression as compared to the Vpx− VLP treated MDMs.
(TIFF)

Figure S3 **HeLa cells were pretreated for 24 h with VLPs prior to infection with either WR or WRΔTK virus (MOI of 0.5 PFU/cell).** At 24 hpi, cells were analyzed for YFP (WR virus) or GFP (WRΔTK virus) expression by flow cytometry. Data were normalized to 1.0 for the Vpx− VLP treatment groups and were graphed. Data shows that HeLa cells are very permissive to vaccinia infection by both WR and WRΔTK viruses.
(TIFF)

Figure S4 **Analysis of PFUs generated by differentiated THP-1 cells.** One million THP-1 cells were differentiated with 50 nM PMA overnight in 6-well dishes followed by VLP treatments. Twenty-four hours later, cells were infected with either WR or WRΔTK virus. Cells and supernatant were collected 24 hpi and then analyzed for PFUs. Data was performed in replicates and plotted.
(TIFF)
