# Supplementary figures and images for: Host Factor SAMHD1 Restricts DNA Viruses in Non-Dividing Myeloid Cells
Source: PLoS Pathog. 2013 Jun 27;9(6):e1003481. doi: 10.1371/journal.ppat.1003481 (PMC3694861; doi:10.1371/journal.ppat.1003481)

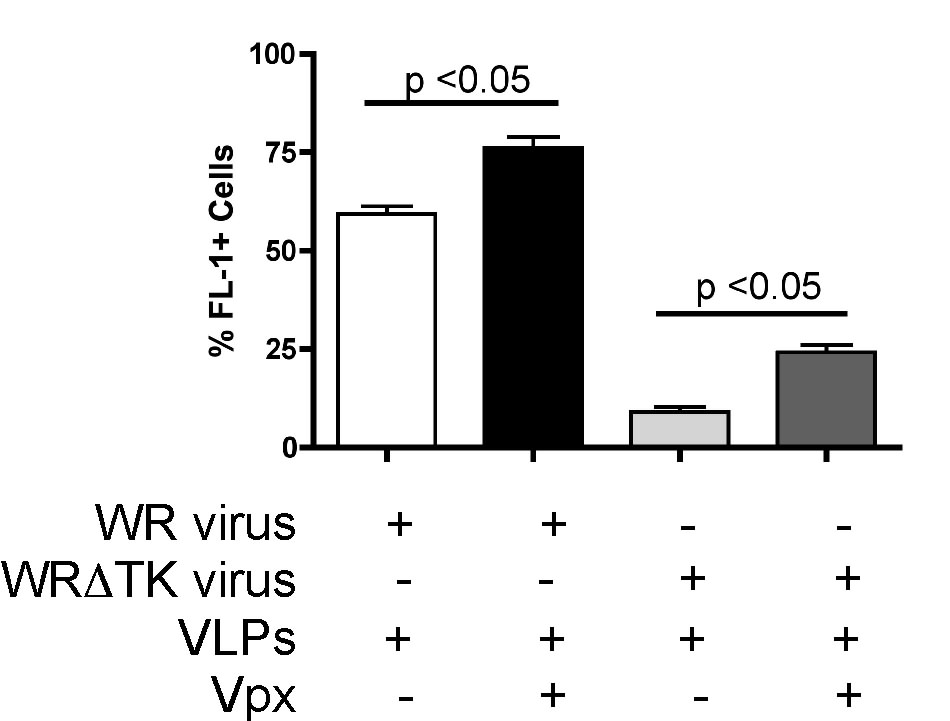

Supplement: Figure S1 — Infection of MDMs donors with different viruses. A total of six MDMs donors were analyzed for the ability of Vpx VLP to enhance the infection of WR and WRΔTK strains. Mann-Whitney test was performed and significant differences indicated with * and p<0.05. (TIFF) [file ppat.1003481.s001.tiff]

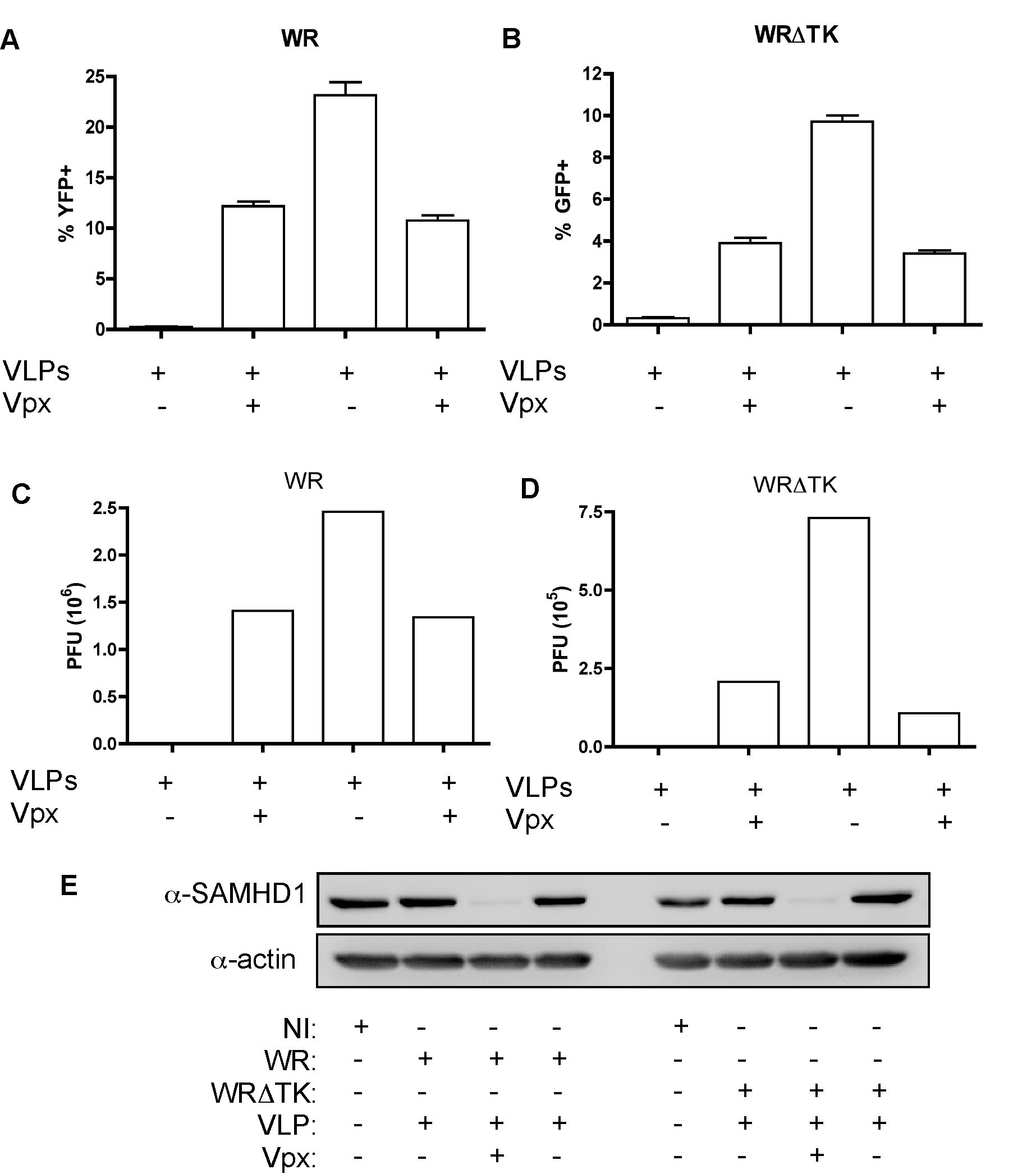

Supplement: Figure S2 — Direct comparison of WR and WRΔTK viruses for one donor. FACS data were plotted for the percentage of (A) WR (YFP+) and (B) WRΔTK (GFP+) cells. PFU for (C) WR and (D) WRΔTK infected MDMs. The non-infected (NI) MDMs had zero PFUs detected. (E) Immunoblot analysis for SAMHD1 expression at 48 h after VLP treatment and 24 hpi. The NI MDMs had comparable SAMHD1 expression as compared to the Vpx− VLP treated MDMs. (TIFF) [file ppat.1003481.s002.tiff]

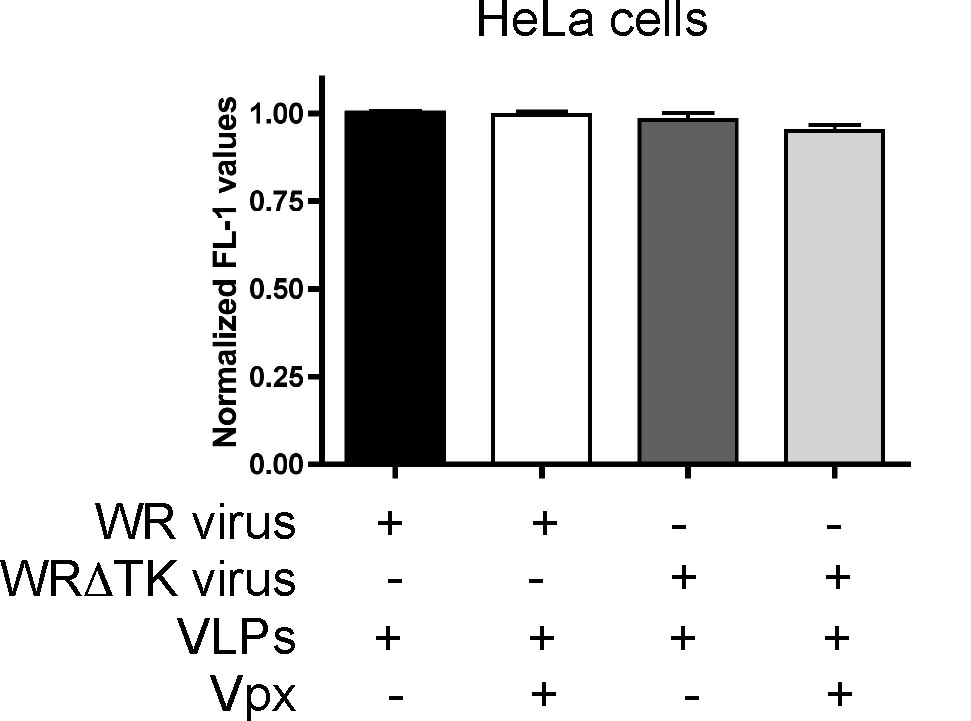

Supplement: Figure S3 — HeLa cells were pretreated for 24 h with VLPs prior to infection with either WR or WRΔTK virus (MOI of 0.5 PFU/cell). At 24 hpi, cells were analyzed for YFP (WR virus) or GFP (WRΔTK virus) expression by flow cytometry. Data were normalized to 1.0 for the Vpx− VLP treatment groups and were graphed. Data shows that HeLa cells are very permissive to vaccinia infection by both WR and WRΔTK viruses. (TIFF) [file ppat.1003481.s003.tiff]

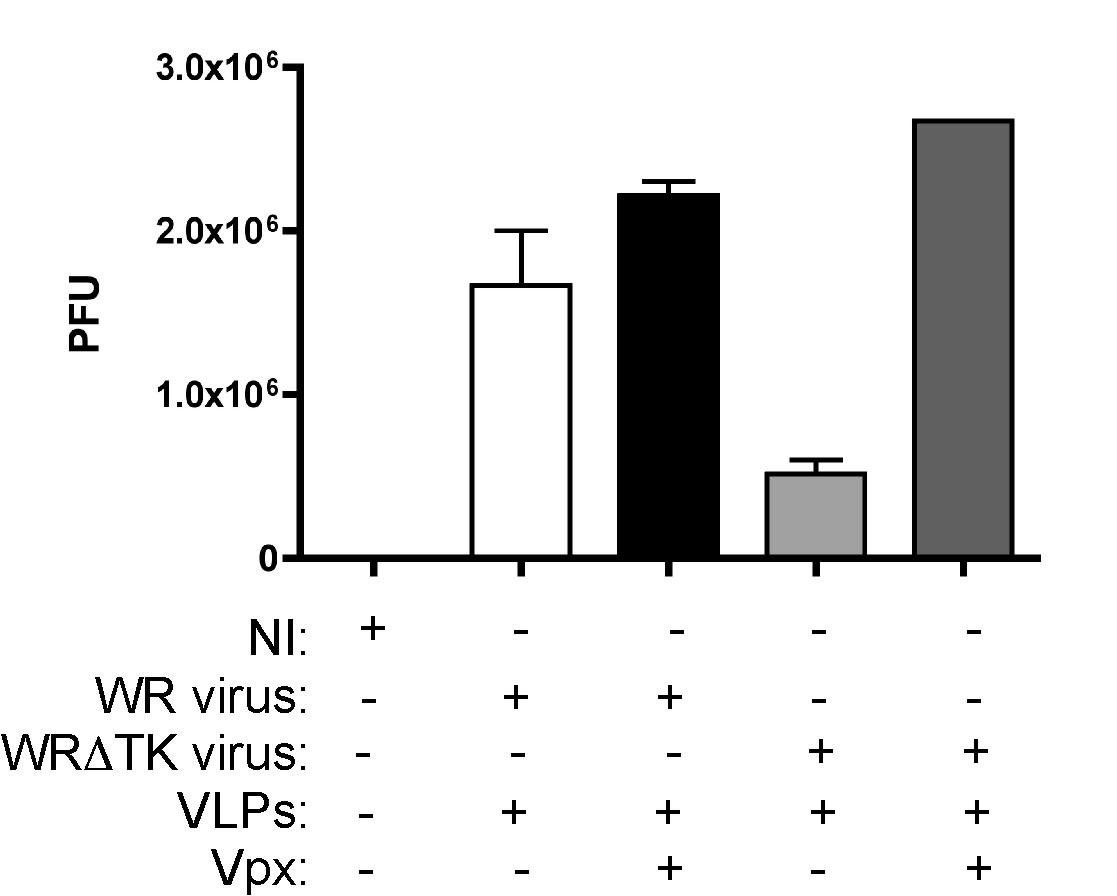

Supplement: Figure S4 — Analysis of PFUs generated by differentiated THP-1 cells. One million THP-1 cells were differentiated with 50 nM PMA overnight in 6-well dishes followed by VLP treatments. Twenty-four hours later, cells were infected with either WR or WRΔTK virus. Cells and supernatant were collected 24 hpi and then analyzed for PFUs. Data was performed in replicates and plotted. (TIFF) [file ppat.1003481.s004.tiff]
